# The molecular characteristics, diagnosis, and treatment of macrolide-resistant *Mycoplasma pneumoniae* in children

**DOI:** 10.3389/fped.2023.1115009

**Published:** 2023-03-02

**Authors:** Maodong Leng, Junmei Yang, Juanjuan Zhou

**Affiliations:** Zhengzhou Key Laboratory of Children's Infection and Immunity, Children's Hospital Affiliated to Zhengzhou University, Zhengzhou, China

**Keywords:** *Mycoplasma pneumoniae*, macrolide resistance, genotype, diagnosis, treatment

## Abstract

The purpose of this study is to review the molecular characteristics, the diagnosis, and treatment of the widespread infection of macrolide-resistant *Mycoplasma pneumoniae* (*M. pneumoniae*; MRMP) in children, thus providing a better knowledge of this infection and presenting the associated problems. Single point mutations in the V region of the 23S rRNA gene of *M. pneumoniae* genome are associated with macrolide resistance. P1–1, MLVA4-5-7-2, and ST3 are usually the predominated genetic types in the *M. pneumoniae* epidemics. The short-term two times serological IgM (or together with IgG) test in the acute stage can be used for confirmation. Combined serological testing and PCR might be a more prudent method to reduce macrolide consumption and antibiotic selective pressure in a clinical setting. Molecular methods for the detection of single-nucleotide mutations in the V region of the 23S rRNA gene can be used for the diagnosis of MRMP. The routine use of macrolide for the treatment of macrolide-sensitive *Mycoplasma pneumoniae* (MSMP) infections can get good effect, but the effects are limited for severe MRMP infections. Additional corticosteroids may be required for the treatment of severe MRMP infections in children in China during the era of MRMP.

## Introduction

*Mycoplasma pneumoniae (M. pneumoniae)* is a main pathogenic cause of community-acquired pneumonia (CAP) and bronchitis, which is responsible for 10%–40% of CAP (1–3). *M. pneumoniae* infection is a kind of self-limited disease, but the infection in some patients, especially in children, can develop serious pneumonia, bronchitis, and asthma, and even endanger their lives. Since there are no intact bacteria in pathologic lesions in severe *M. pneumoniae* pneumonia (MPP) patients and experimental animals in species-specific mycoplasma strains, the pathogen itself may not be a direct cause of cell injury in *M. pneumoniae* infections, and the theory of immunopathogenesis is proposed. The overuse and unnecessary usage of macrolides for the treatment of respiratory infections in recent years have contributed to the increase of macrolide-resistant *M. pneumoniae* (MRMP). Since MRMP was first isolated and described in pediatric patients in Japan (4), it spread rapidly all over the world, especially in Asian countries including China, Japan, and South Korea, where the resistance rates could reach 80%–90% (5–7). Studies found that single-nucleotide mutations in the V region of the 23S rRNA gene of *M. pneumoniae* genome are associated with macrolide resistance (8), and A2063G is the most common mutation, followed by A2064G. Clinical isolates of *M. pneumoniae* can be typed by *p1* adhesion gene typing (9), multiple-locus variable-number tandem-repeat analysis (MLVA) (10, 11), and multilocus sequence typing (MLST) (12, 13). Common methods for the detection of *M. pneumoniae* include culture, serum special anti-*M. pneumoniae* antibody tests, and molecular methods. PCR or sequencing methods could be performed to identify the macrolide resistance-associated mutations within the V region of 23S rRNA gene of the *M. pneumoniae* genome. Macrolide is regarded as the first-line antibiotic treatment for *M. pneumoniae* infections in children for its low minimum inhibitory concentrations (MICs) and toxicity. Treating *M. pneumoniae* infections is more difficult in China because of the high macrolide-resistant rates. The routine use of azithromycin for the treatment of macrolide-sensitive *M. pneumoniae* (MSMP) infections can achieve a good effect, but additional corticosteroids may be required for the treatment of MRMP infections. Corticosteroid use has been reported in children of South Korea infected with MRMP or MSMP, which showed reduced disease morbidity and disease progression in MPP patients without side effects (14). Although some achievements have been made, there are some unsolved issues about MRMP. The pathogenesis of MPP and its complications remain unknown; the molecular characteristics of *M. pneumoniae* including the genotypes and macrolide resistance-associated single-nucleotide mutations are unclear in many parts of China and the world, and the correlation between macrolide resistance and genotypes remain controversial; the early and definite diagnosis of *M. pneumoniae* infection and the macrolide resistance is difficult, along with the proper additional corticosteroids treatments for MRMP infections.

## Etiology and pathophysiology

*M. pneumoniae* is a kind of small atypical prokaryotic pathogen, with body size lager than a virus but smaller than a bacterium, and it can go through a bacterial filter and cannot be identified under an optical microscope. However, *M. pneumoniae* is considered a self-replicating bacterium for its ability to live outside a host cell and in the medium, and it can be inhibited or killed by antibiotics in patients with lower respiratory infections (15, 16). The growth of *M. pneumoniae* is very slow; thus, up to 6 weeks are required for the culture of *M. pneumoniae* (17). *M. pneumoniae* has a small genome of approximately 800 kb in size and up to 700 protein-coding genes (18–20), and the genome appears to be highly conservative between the strains (21). *M. pneumoniae* lacks a cell wall, and the special structure makes *M. pneumoniae* resistant to *β*-lactam antibiotic instinctively and facilitates the membrane of *M. pneumoniae* contact with that of the host cell directly, thus transferring and exchanging membrane components (22). *M. pneumoniae* is a unique human pathogen and can be transmitted through droplets between humans, and it can be transmitted among family members who live together in a house or among students with close and frequent touches in a school (23, 24). *M. pneumoniae* infection occurs both endemically and epidemically worldwide and it can induce upper and lower respiratory infections (21). *M. pneumoniae* infection can occur at any time during a year but may be more common in the summer and early autumn; an epidemic cycles at a time interval of 3–7 years (25), and each epidemic may last 1–2 years. Meteorological factors play an important role in the incidence of *M. pneumoniae,* and the infection rate may be positively correlated with temperature (26). The symptoms of *M. pneumoniae* infections include sore throat, fatigue, fever, headache, and cough that can last for weeks to months. Infected children younger than 5 years old are unlikely to develop fever but may manifest as wheezing, vomit, diarrhea, stuffy or runny nose, and sneezing (25). Extrapulmonary manifestations may be present other than the respiratory infections, affecting the skin mucous membrane, liver, kidney, and central nervous systems, such as the “*Mycoplasma*-induced rash and mucositis” (27).

The self-limited nature of *M. pneumoniae* infection often results in mild symptoms or being asymptomatic, but it may sometimes lead to dysfunction of the localized organs. The pathogenic mechanisms of *M. pneumoniae* include (1) activating the innate immune response and producing local cytotoxic effects through a specialized attachment to host cells and (2) leading to inflammation and airway dysfunction through producing a unique virulence known as Community-Acquired Respiratory Distress Syndrome (CARDS) toxin (25). The pathophysiology of acute lung injury and extrapulmonary manifestations, such as skin rashes, arthritis, encephalopathy, and other organ cell injuries on *M. pneumoniae* infection or coronavirus disease 2019 (COVID-19) are not fully understood. It is proposed that *M. pneumoniae* may act like a virus in the pathophysiology of the disease (28). Thus, it is possible that inflammation-inducing substances in *M. pneumoniae* infection are produced when pathogens are replicated within host cells like viruses. Host immune system may control these etiologic substances that originate from pathogens, including toxins such as CARDS toxin, and pathogen-associated molecular patterns (PAMPs), which lead to the release of pro-inflammatory cytokines. The substances originated from injured infected-host cells called damage-associated molecular patterns (DAMPs) can result in the elevation of inflammatory cytokines levels. The protein-homeostasis-system hypothesis proposes that the immune systems in the host control the etiological substances in *M. pneumoniae*-infected cells like viruses according to the size and biochemical properties (29). When these substances spread systemically and locally and bind to target organ cells, pneumonia and extrapulmonary manifestations begin due to the activation of corresponding immune cells and immune proteins. The substances produced from injured target cells such as lung cells induce further inflammation if released into the systemic circulation or near local lesions, and they could be associated with extrapulmonary manifestations (30).

## Macrolide resistance

As a kind of bacteriostatic agent, macrolide antibiotics act by inhibiting protein synthesis of bacteria through binding to the 50S ribosomal element (31). Macrolide is regarded as the preferred antibiotic choice for the treatment of *M. pneumoniae* infections than tetracyclines or quinolones especially in children because of the toxicities of the latter two. *In vivo* studies found that macrolide resistance-associated mutations including A2063G and A2064G could be found in 100% patients after 7–24 days of the initiation of macrolide treatment (32), and *in vitro* studies showed that the resistance mutations could be induced by subinhibitory concentrations of macrolide (33). Therefore, the overuse and unnecessary usage of macrolide in the past years are the main reasons for the prevalence of MRMP.

The resistance mechanisms of *M. pneumoniae* are mainly associated with the alteration of action targets of macrolide antibiotics. It has been identified and confirmed that the single-nucleotide mutations in domain V of the 23S rRNA gene are responsible for the macrolide resistance of *M. pneumoniae* (8, 34, 35). The mutations at the V region of the 23S rRNA gene decrease the affinity of macrolide to the 23S rRNA gene, and the ability of macrolides to suppress the protein synthesis of *M. pneumoniae* weakens, thus resulting in reduced strength of macrolides to inhibit the growth of *M. pneumoniae*. The mutations at sites 2063, 2064, 2067, and 2617 of 23S rRNA gene are the frequent mutations that confer macrolide resistance (9, 10, 13, 36–38), and mutations at sites 2063 and 2064 are associated with a high level of resistance, whereas the 2067 and 2617 site mutations result in a low level of resistance (35, 39). MRMP has very high MICs for 14- and 15-membered ring macrolides and moderately high MICs for 16-membered ring macrolides, and strains with the A2063G mutation have lower MICs for rokitamycin and josamycin (40).

The rates of MRMP vary across different regions of the world. The prevalence of MRMP is shown in Table 1 (11–13, 37, 41–47), and we selected and summarized the representative studies from regions of the world including America, Japan, South Korea, China, and Europe. From Table 1, it can be concluded that the prevalence of MRMP from high to low among different regions are northeastern Asia, America, and Europe. The rate of MRMP in the United States is about 10%, and it is often below 10% in Europe, whereas the rate in Asia is far higher. A2063G of the V region of the 23S rRNA gene is nearly the most frequently identified macrolide resistance-associated mutation in all regions, followed by A2064G. The data about MRMP are concentrated in America, Japan, South Korea, and European countries, and in Beijing and Shanghai of China. The lack of data about macrolide resistance in many parts of the world and China may be due to technical difficulties and cost reasons. There is no significant difference in clinical manifestation and disease severity between patients infected with MRMP and MSMP, but patients infected with MRMP may display prolonged hospitalization, febrile and coughing days, and antibiotic treatment course; more MRMP-infected patients experienced a change of antibiotic prescription under the traditional antibiotic treatments (48–50). Children with MRMP infections could appear with higher leukocyte counts and C-reactive protein (CRP) compared with patients infected with MSMP (51), but other studies suggested that MRMP is unlikely to be associated with laboratory and radiographic severity (48, 52); the contradictions might have resulted from the different subjects selected or the different time points in which the examinations were performed.

**Table 1:** The macrolide resistance and genotypes of *M. pneumoniae.*

Country or district	Year	Cohort	Number of cases	Macrolide resistance rate (%)	Resistance-associated mutation types	Typing methods	The most prevalent reported types	Macrolide resistance and genotypes	References
America	2006–2013		199	10		P1 and MLVA	P1–1 and MLVA4-5-7-2	No correlation observed	(41)
America	2012–2018		446	8.3	Mainly A2063G	P1 and MLVA	P1–2 and MLVA3-5-6-2	No correlation observed	(42)
Japan	2011–2017		419	50.1	A2063G (main) and A2063T	P1	P1–2	P1–1 with MRMP, P1–2 with MSMP	(43)
Japan	2002–2016	Children and adults	45 adults and 372 children	44.4	A2063G (main), A2063T, A2064G, A2063C, and C2617A	MLST	ST3	ST3 and ST19 with MRMP	(37)
South Korea	2000–2016	Children	146	0–84.4	A2063G and A2064G	MLST and P1	ST3 and P1–1	ST3 with MRMP	(13)
Beijing	2003–2015	Children	480	94.8	A2063G (main), A2064G, and A2063T	P1 and MLVA	P1–1 and MLVA4-5-7-2	MLVA4-5-7-2 and P1-1 with MRMP	(11)
Beijing	2016	Children	214	66	A2063G (main) and A2064G	MLVA	MLVA4-5-7-2	MLVA4-5-7-2 with MRMP	(44)
Taiwan	2016	Children	180	24	A2063G (main), A2063T, and A2064G	MLVA	MLVA4-5-7-2	MLVA4-5-7-2 with MRMP	(45)
Taiwan	2017–2019	Children	226	74	A2063G (main), A2063T, and A2064G	MLST	ST3	ST3 and ST17 with MRMP	(12)
Spain	2013–2017		137	8	A2063G mainly	MLVA	MLVA4-5-7-2	MLVA4-5-7-2, 3-5-6-2 with MRMP	(46)
Switzerland	2016–2020	Children	54	1.9	A2063G	P1, MLVA and MLST	P1–1 and P1–2, MLVA3-5-6-2, and ST14		(47)

M. pneumoniae, Mycoplasma pneumoniae; MLVA, multiple-locus variable-number tandem-repeat analysis; MRMP, macrolide-resistant M. pneumoniae; MSMP, macrolide-sensitive M. pneumoniae; ST, sequence type.

## Genotyping

The most frequently performed methods for the genetic typing of *M. pneumoniae* include *p1* adhesion gene restriction fragment length polymorphism (RFLP) analysis, MLVA, and MLST. The gene *p1* is a kind of adhesion gene, which is the major factor that determines the virulence of *M. pneumoniae* (53). The P1 protein can cause allergy by inducing the production of P1-specific IgE (54). RepMP2/3 and RepMP4 are two repeated elements of the *p1* gene, and *M. pneumoniae* strains can be classified into two major types (P1 subtype 1 and subtype 2, P1–1 and P1–2) based on the RFLP of the two regions (55–57). The regions can be combined with similar regions outside the *p1* gene, thus generating the V1, V2a, V2b, V2c, and V2d variants (58). MLVA includes the analysis of five tandem repeated regions, which are named Mpn1, Mpn13, Mpn14, Mpn15, and Mpn16. Due to the instability of Mpn1, it might be more reliable to use the four-locus analysis (excluding Mpn1) to identify *M. pneumonia* (59). MLVA may be only useful for the identification or comparison of strains that are collected from relatively limited area and over a short period (60). MLST is performed through the sequencing of the eight housekeeping genes (*ppa*, *pgm*, *gyrB*, *gmk*, *glyA*, *atpA*, *arcC*, and *adk*) of *M. pneumoniae*, and *M. pneumoniae* can be typed based on the polymorphisms of the house keeping genes (61). MLST has higher discriminatory power than the frequently used four-locus MLVA typing and *p1* gene RFLP methods (23).

The typing methods, the most prevalent reported types, and the correlations between different genetic types and macrolide resistance in studies are shown in Table 1. According to Table 1, P1–1 type predominated in most districts and periods, except the P1–2 predominance during 2012–2018 in America and during 2011–2017 in Japan, and the equal predominance of P1–1 and P1–2 during 2016–2020 in Switzerland. The two studies in America revealed a type shift from P1–1 to P1–2, which might be due to changes of human immunity, such as during the prevalence of P1–1, the human immunity against it strengthened (60). From Table 1, it can be summarized that MLVA4-5-7-2 and MLVA3-5-6-2 are the most prevalent reported MLVA types. In the two American studies, the MLVA types changed from MLVA4-5-7-2 during 2006–2013 to MLVA3-5-6-2 during 2012–2018, which revealed a MLVA type shift. ST3 predominated in most studies except the ST14 predominance in Switzerland from 2016 to 2020. The prevalence of MRMP and the genotypes between children and adults may be different, and the study by Yan et al. demonstrated that more diverse genotypes and a higher prevalence of macrolide resistance-associated mutations were found in the pediatric specimens (62). Further studies are needed to explore the molecular differences of *M. pneumoniae* infections between adult and pediatric population.

Controversial opinions existed regarding the correlations between different genetic types and macrolide resistance, and final conclusion has not been achieved yet. According to Table 1, the correlation between different genetic types and macrolide resistance can be established in Asian countries such as China, Japan, and South Korea, whereas few correlations existed in America and European countries, which might be due to the higher prevalence of MRMP in China and Japan whereas the lower prevalence of MRMP in America and European countries. In China, Japan, and South Korea, the prevalence of P1–1, ST3, and MLVA4-5-7-2 types are always correlated with MRMP (63). Periodic genotype shifts of the *p1* gene in Japan from 2006 to 2019 found a decreased rate of MRMP and P1–1 type simultaneously (60), which can be speculated that the reduction of MRMP might be caused by the prevalence of P1–2-type *M. pneumoniae*. Controversial opinions suggest that the prevalence of MRMP is correlated with the use of macrolides but not genotypes for the different macrolide resistance rates of P1–1-type *M. pneumoniae* across different regions (42).

## MRMP diagnosis

The methods for the diagnosis of *M. pneumoniae* include culture, PCR assays, and serologic tests. The advantages and disadvantages of each diagnostic method are shown in Table 2. The PPLO (pleuropneumonia-like organisms) broth supplemented with nutrients is often used for the culture of *M. pneumoniae*. The growth of *M. pneumoniae* is slow, and the diagnosis is time consuming, so it is not suitable for the early diagnosis and treatment of *M. pneumoniae* infection. On the other hand, the culture of *M. pneumoniae* requires high conditions of medium, and thus the positive rate is low. PCR assays include the detection of DNA and RNA, and the most frequently used method for the detection of DNA is the real-time PCR targeting the adhesion *p1* gene of *M. pneumoniae* (64). The median duration of persistence of *M. pneumoniae* DNA in the body is 7 weeks after the onset of *M. pneumoniae* infection disease, and the period cannot be shortened even if adequate antibiotic treatment is given (65). Therefore, the *M. pneumoniae* DNA can be positive even after the symptoms of *M. pneumoniae* infection disappear, whereas *M. pneumoniae* RNA can be cleared quickly after the death of *M. pneumoniae*, and the detection of RNA can be negative after the recovery of the infection symptoms. The problem of the false-positive possibility of *M. pneumoniae* infection due to the persistence of *M. pneumoniae* DNA can be avoided by using RNA detection. *M. pneumoniae* RNA detection is capable of distinguishing recent and past infections; thus, it can be used to evaluate the therapeutic effect and the prognosis of the disease (66). There are many asymptomatic infected patients during *M. pneumoniae* epidemics and some co-infected patients. Thus, carriers and co-infected patients can be PCR positive (false positive), and it is necessary to identify the asymptomatic infection of *M. pneumoniae* and distinguish the real cause of symptoms. A serological test is the most widely used method for the diagnosis of *M. pneumoniae* infection currently. The main targets for the serological test are the special anti-*M. pneumoniae* IgG and IgM. The production of IgM requires 1–2 weeks, and it can exist positively a long time of over 1 year. A sensitivity of approximately 70%–80% and a specificity of 90% could be achieved by the perfect IgM test (67). The sensitivity of *M. pneumoniae* IgM test is higher than that of IgA for the diagnosis of *M. pneumoniae*-related pneumonia in school-age children and adolescents, and it is interesting to find that the rates of IgM and IgA are positively associated with the febrile days before hospitalization (68). In a clinical setting, because the PCR results much depend on the specimen collection process, PCR-positive patients at presentation are far less than single IgM-positive patients, and PCR-negative patients, especially lately presented, are common over 20%–50% of study subjects (69). There are no confirmative diagnostic laboratory tools for *M. pneumoniae* infection in the early stage, and single IgM-positive and/or single PCR tests are not confirmative for diagnosis of *M. pneumoniae* infection. Paired sera tests could provide a more accurate diagnosis of *M. pneumoniae* infection, and the fourfold increase of IgG from the acute to the recovery stage is regarded as the gold standard (70). The positive rates of paired sera IgG tests could be over 80% (71). The limitation of paired sera IgG test is that it can be only used for retrospective diagnosis and could not be applied for the early diagnosis. The long-time existence of IgM in the body can result in false positivity, and IgM serology may lead to false positive due to limited assay performance and age/host-dependent characteristics or false-negative results early in disease course and after reinfection (72). Chang et al. demonstrated that IgM showed poor sensitivity and positive predictive value, so the interpretation of IgM should be done with caution (73). Serological test only reflects the host immune response to *M. pneumoniae* (69). Serological changes, including IgM, IgG, and IgA, begin to appear after clinically manifesting such as fever and pneumonia. Thus, short-term two times serological IgM (or together with IgG) test in the acute stage is needed for confirmation of *M. pneumoniae* infection, and its clinical application is easy during hospitalization (74). Considering the advantages and disadvantages of serological and PCR tests, combined serological testing and PCR might be a more prudent method to reduce macrolide consumption and antibiotic selective pressure in a clinical setting.

**Table 2:** The advantages and disadvantages of each diagnostic method of *M. pneumoniae.*

	Advantages	Disadvantages
Culture	Direct evidence of *M. pneumoniae* infection.	Not suitable for the early diagnosis and the positive rate is low.
Serological	Convenient, accurate and efficient, and can be used for confirmation test.	False-negative and false-positive possibilities.
Molecular methods	Accurate, simple, fast and easy to practice. RNA test can be used for the current infection diagnosis.	False positivity of DNA test, the low positive rate of DNA and RNA tests in clinical settings.

M. pneumoniae, Mycoplasma pneumoniae.

The macrolide resistance of *M. pneumoniae* can be determined by the drug susceptibility experiment and molecular methods currently. Drug susceptibility experiment based on the broth microdilution method can not only determine the macrolide resistance of *M. pneumoniae* but also test the exact minimum inhibitory concentration. However, drug susceptibility experiment requires the culture of *M. pneumoniae*, so it is not suitable for the clinical practice due to the long process to the final diagnosis. Molecular methods for the detection of single-nucleotide mutations in the V region of the 23S rRNA gene of *M. pneumoniae* can be used for the rapid diagnosis of MRMP. The most widely used molecular method for the detection of MRMP can be performed by the amplification of the V region of the 23S rRNA gene; the products are then purified and sequenced, and finally the sequence results are blasted with the corresponding sequence of the reference standard strain M129 signed in NCBI to identify the nucleotide mutations associated with macrolide resistance. Coexistence of MRMP and MSMP can be found in a single case (75), and the determination of the ratio of MRMP/MSMP may be valuable for the diagnosis. Pyrosequencing is the only method for the quantification of MRMP and MSMP within a clinical specimen (76). Melting curve analyses based on PCR assay could reliably distinguish MRMP isolates when compared to MRMP and MSMP controls included in the run due to the slightly higher melting temperature of the amplicon caused by the substitution of A to G at positions 2063 or 2064(77). The melting curve analysis can be an efficient method to identify macrolide resistance, and the analysis can be completed within 1 h (76). The melting curve analysis can be practical for the fast identification of the A2063G and A2064G transitions, which usually cover over 90% of macrolide resistance mutations, and these two kinds of mutations usually confer high-level macrolide resistance, representing valuable clinical significance.

## MRMP treatment

*M. pneumoniae* is instinctively resistant to *β*-lactam antibiotics, and macrolides, tetracyclines, and fluoroquinolones are effective selections for the treatments. Considering the risks of the antibiotics for the human body, macrolides can be used for both children and adults, tetracyclines can be used for elder children and adults, and fluoroquinolones for the adults only. The emergence of MRMP infections has made the antibiotic treatment more complicated. Macrolide possesses anti-inflammatory and immunoregulatory functions, enabling it effective in the treatment of some mild MRMP-infected cases. For severe cases infected with MRMP that showed poor effects during the treatments with macrolides, alternative antibiotic treatments have been explored. Japan Society of Pediatric Pulmonology/Japanese Society for Pediatric Infectious Diseases published guidelines for recommending tosufloxacin as a second-line drug when patients remain febrile for 48–72 h following the administration of macrolides (78). Doxycycline has been used as an alternative and is not likely to cause visible teeth staining or enamel hypoplasia in young children for short periods of treatments (79). An attempt of treatment of MPP cases infected by MRMP with levofloxacin has been made, which proved to be safe and effective, the clinical symptoms and radiological manifestations improved significantly, and no side effects were observed (80). There may be a long way for the exploration of safe and effective alternative antibiotic treatments, and surveillance should be given to the possible emergence of clinical tetracyclines or fluoroquinolones resistant strains, due to the fact that resistant strains have been induced *in vitro* for both antibiotic with the associated mutations (81–83).

Since MPP, including refractory MPP or MRMP pneumonia, is a self-limited disease, the host hyperimmune reaction against insults from *M. pneumoniae* infection is responsible for lung cell injury. The immune reaction of the host before the peak of inflammation (pro-inflammatory cytokines may be involved in this stage) may be involved in tissue cell injury, and immune reaction after the peak of inflammatory may be involved in tissue cell repair (anti-inflammatory cytokines may be involved in the convalescent stage); the intensity of systemic inflammation during this process is reflected in laboratory parameters, such as WBC, and differential, CRP, LDH, and immune proteins, such as IL-6 and other cytokines and chemokines. The interleukin-6/interleukin-10 ratio is an effective biomarker for discriminating *M. pneumoniae* pneumonia from respiratory syncytial virus pneumonia (84). The severity of lung injury in *M. pneumoniae* infection may be associated with the extent of host immune reaction against the amount of etiologic or inflammation-inducing substances in the acute stage. Thus, early control of this process is critical in immune modulator (corticosteroid) treatment of *M. pneumoniae* infection (85). Also, early control of lung injuries from initial hyperactive immune reactions is crucial for reduction of morbidity and prevention of pneumonia progression and complications in patients with MPP94. In the era of macrolide-resistant *M. pneumoniae* strains and based on immunopathogenesis of *M. pneumoniae*, now early immune modulators, including corticosteroids, are reliable to use for *M. pneumoniae* (86, 87). The effect of immune modulators (corticosteroid and/or intravenous immunoglobulin) on immune cells is also dose-dependent, and the dose of corticosteroid use can be adjusted according to severity of the disease. Thus, it is possible that patients with severe MPP have more severe immune disturbance and respond to the higher-dose immune modulators because of same immune pathogenesis of mild and severe MPP. The open problem now is the optimal time, dose, and schedule of immune modulator therapy based on the severity of the disease and the need for alternative antimicrobial drugs may not be that urgent (88).

## Conclusion

*M. pneumoniae* infection is a main cause of CAP in children, and MRMP spreads as a result of the overuse and unnecessary usage of macrolides. The pathogenic mechanism of *M. pneumoniae* infection is mainly associated with the hyperimmune response of the host to the substances from the infection, thus causing lung injury and extrapulmonary manifestations. Macrolide resistance of *M. pneumoniae* is mainly associated with nucleotide transition mutations in domain V of the 23S rRNA gene, and A2063G is the most prevalent mutation, followed by A2064G. P1–1, MLVA4-5-7-2, and ST3 are usually the predominated types in the *M. pneumoniae* epidemics, but genotype shifts may occur through different time periods. The correlation between genotypes and MRMP still remains controversial. Short-term two times serological IgM (or together with IgG) test in the acute stage can be used for confirmation. The identification of MRMP can be performed by the detection of single-nucleotide mutations within the 23S rRNA gene. The routine use of macrolide for the treatment of MSMP infections can get good effect, but the effects are limited for severe MRMP infections. Early immune modulator (corticosteroid and/or intravenous immunoglobulin) treatment is crucial for controlling the immune reaction-associated cell injuries, thus reducing morbidity and preventing pneumonia progression and complications. Additional corticosteroids may be required for the treatment of severe MRMP infections in children of China during the era of MRMP.
